# Does the Minerals Content and Osmolarity of the Fluids Taken during Exercise by Female Field Hockey Players Influence on the Indicators of Water-Electrolyte and Acid-Basic Balance?

**DOI:** 10.3390/nu13020505

**Published:** 2021-02-04

**Authors:** Joanna Kamińska, Tomasz Podgórski, Krzysztof Rachwalski, Maciej Pawlak

**Affiliations:** 1 Chair of Dietetics, Department of Physiology and Biochemistry, Poznań University of Physical Education, 61-871 Poznań, Poland; podgorski@awf.poznan.pl (T.P.); pawlak@awf.poznan.pl (M.P.); 2 Chair of Theory and Methodology of Sport, Department of Theory and Methodology of Team Sport Games, Poznań University of Physical Education, 61-871 Poznań, Poland; rachwalski@awf.poznan.pl

**Keywords:** hydration status, water–electrolyte balance, acid–base balance, fluids osmolarity, team sports, nutrition, women in sport

## Abstract

Although it is recognized that dehydration and acidification of the body may reduce the exercise capacity, it remains unclear whether the qualitative and quantitative shares of certain ions in the drinks used by players during the same exertion may affect the indicators of their water–electrolyte and acid–base balance. This question was the main purpose of the publication. The research was carried out on female field hockey players (*n* = 14) throughout three specialized training sessions, during which the players received randomly assigned fluids of different osmolarity and minerals contents. The water–electrolyte and acid–base balance of the players was assessed on the basis of biochemical blood and urine indicators immediately before and after each training session. There were statistically significant differences in the values of all examined indicators for changes before and after exercise, while the differences between the consumed drinks with different osmolarities were found for plasma osmolality, and concentrations of sodium and potassium ions and aldosterone. Therefore, it can be assumed that the degree of mineralization of the consumed water did not have a very significant impact on the indicators of water–electrolyte and acid–base balance in blood and urine.

## 1. Introduction

Field hockey is a team sport practiced by women and men, both on a recreational and professional level [1]. At the elite level, field hockey players cover between 3.4 km and 9.5 km during training and competition depending on their position on the pitch [2,3], with 55% being low-intensity efforts (standing, walking), 38% moderate intensity (jogging, running), and the remaining 7% being high-intensity efforts (fast running, sprinting) [4]. Physical activity increases the body’s internal temperature [5]. Compensating mechanisms, especially increased sweat production [6], cause a loss of water and the electrolytes contained in it [7]. Moreover, acidic compounds formed during exercise simultaneously contribute to disturbances in the acid–base balance [8,9], reducing sports performance, especially muscle endurance [10] or cognitive functions [11]. It is therefore important to control and, if necessary, replenish fluids of an appropriate qualitative and quantitative profile before or during exercise. The transport of fluids into the bloodstream and tissues depends, *inter alia*, on the speed at which they leave the stomach and on the effectiveness of their absorption in the small intestine. The above depends on the volume of fluid consumed, the content of energetic substances such as glucose in the fluid, and the concentration gradient on both sides of the intestinal barrier. Thus, the transport of water from the intestines into the bloodstream and tissues is greater for hypotonic than hypertonic drinks, since the latter support the movement of water from the tissues into the lumen of the gastrointestinal tract [12]. On the other hand, the ingestion of low-mineral water (hypotonic solutions) reduces plasma osmolality, which stimulates urine production and may increase dehydration [13,14].

The regulation of the concentration of sodium and potassium ion levels in the body depends on the renin–angiotensin–aldosterone system (RAA). Aldosterone inhibits the excretion of sodium in the urine, increasing the loss of potassium ions from body fluids. In addition, this hormone is secreted as a result of an increase in the concentration of hydrogen ions in the blood accompanying exercise [15]. Such an excess of hydrogen ions is excreted in the urine, and the speed of this process depends on the amount of urine excreted.

The volume of fluids consumed by humans is regulated not only by physiological factors, but also by subjective ones, whereby the taste attractiveness of liquids may prove to be an important factor in the hydration process, especially taking into account the volumes consumed by players [16]. In the case of water, its tastiness is influenced by an increase in the proportion of sodium ions, which stimulates physiological thirst and thus leads to better hydration [13]. Therefore, isotonic fluids most effectively compensate for water and electrolyte losses caused by physical exercise, while providing additional energy [17].

It has been hypothesized that consuming beverages with a higher osmolarity and/or content of minerals lead to a more favorable water–electrolyte and acid–base balance compared to the intake of low-mineralized water. To verify this hypothesis, taking into account the intensity and duration of training, ambient conditions and the athlete’s individual physiological profile, during the standard training of female field hockey players, was the main purpose of the research. In the publication, the authors also set themselves the goal of describing changes in the water–electrolyte and acid–base balance indicators in terms of adapting the bodies of female field hockey players to standard training loads.

## 2. Materials and Methods

### 2.1. Experimental Approach

The levels of biochemical and hematological indicators in female field hockey players were assessed during three specialized training sessions, during which the players randomly received fluids of different osmolarities. The water–electrolyte and acid–base balance was assessed on the basis of blood and urine indicators, which were obtained immediately before and after each training session.

### 2.2. Participants

The research included 14 players training in field hockey, members of the women’s national team and women’s junior team. Players participated in training sessions five times per week. The weekly training time was, on average, 6.5 ± 1.0 h. Training was complemented with 1 match per week during the three weeks of the research period. The weekly framework of the training program is presented below:

Monday—active recovery training + static stretching
Tuesday—technical/tactical training session + interval run
Wednesday—technical/tactical training session (research measurements)
Thursday—training game/small side games
Friday—individual gym session
Saturday—free/passive recovery
Sunday—Polish League competition

Goalkeepers were excluded from the research due to the different nature of the effort they performed.

The average training experience of the female competitors was 12.5 ± 2.9 years. They were non-smokers, as evidenced by the mean contents of carboxyhemoglobin in the blood of female hockey players, amounting to 0.9% ± 0.2%. For 22 h before the tests, the contestants did not perform any intense physical effort. An hour before each test date, they ate a standard meal (porridge in milk with banana), the caloric content of which corresponded to 10% of the daily food ration of each of the competitors. Additionally, the competitors were not allowed to eat any food during the whole training.

**Figure 1:**
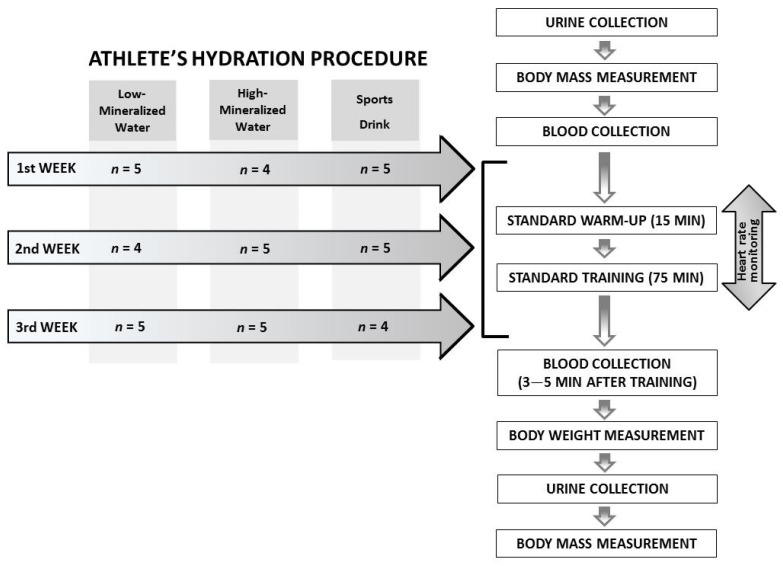
Flow chart of the study design.

The anthropometric data of the players were determined on the basis of measurements made before the start of each training session (Table 1). Their height and weight were measured using a medical scale WPT60/150 OW (Radwag^®^, Radom, Poland), while the waist circumference was measured using a tailor’s tape measure. The mass of urine excreted was determined by comparing body mass immediately after exercise and body mass after urination (Figure 1).

### 2.3. Ethics Approval

The research related to human use complied with all relevant national regulations and institutional policies, has followed the tenets of the Declaration of Helsinki, and has been approved by the Bioethical Committee of the Poznan University of Medical Sciences (Approval No.: 140/15).

Informed consent was obtained from all individual participants included in the study.

### 2.4. Biochemical Analyses

The material for the research was capillary blood obtained from the fingertip of the non-dominant hand of the players, before and after the standard training unit. Blood was collected according to the applicable procedures, from the finger of the non-dominant hand using a Medlance^®^ Red lancet-spike (HTL-Zone, Berlin, Germany) with a 1.5 mm blade and 2.0 mm penetration depth. In addition, each of the contestants was asked to submit a urine sample before and after training.

In blood collected from a heparinized capillary (65 μL), the concentration of electrolytes (Na^+^, K^+^, Ca^2+^, Cl^−^, HCO_3_^−^), lactate, plasma osmolality and pH, and standard base excess (BE), were determined using a gasometric analyzer (ABL90 FLEX, Radiometer, Copenhagen, Denmark). Additionally, 300 µL of capillary blood was collected in a Microvette^®^ CB 300 tube (Sarstedt, Nümbrect, Germany) containing K2-EDTA (EDTA dipotassium salt) as an anticoagulant for hematocrit determination on a hematology reader (Mythic^®^18, Orphèe, Geneva, Switzerland). Another 300 µL of capillary blood was collected in a Microvette^®^ CB 300 Z tube (Sarstedt, Nümbrect, Germany) with a clotting activator, in which the concentration of aldosterone was determined using an ELISA kit (DRG MedTek, Warsaw, Poland; Cat No. EIA-5298) and magnesium using the colorimetric method (Mg; Cormay, Łomianki, Poland; Cat No. 2-229). The absorbance readings were taken on a multi-detection microplate ELISA reader (Synergy 2 SIAFRT, BioTek, Winooski, VT, USA). Urine-specific gravity and pH were determined on a urine strip analyzer (URYXXON^®^ Relax, Macherey-Nagel, Düeren, Germany).

### 2.5. Specialized Training

The three test dates were carried out at weekly intervals, in November and December 2016, in the hall, each time from 6:00 to 7:30 p.m. Each time, the contestants were randomly assigned to groups consuming fluids with different osmolality levels in a manner ensuring the consumption of each of the drinks (Figure 1). The competitors had free access to the randomly drawn fluid during each of the 1.5 h training sessions (Table 2) and decided on both the time and amount of intake (Table 1). The composition of the liquids, given by the producers on the packaging, is presented in Table 3, and the osmolarity of these beverages was adopted based on the available literature: low-mineralized water ~20 (mOsm/kg water), highly mineralized water ~88 (mOsm/kg water), and isotonic drink ~279 (mOsm/kg water) [18].

During each training session, the air temperature and humidity were measured using data loggers located in the four corners of the pitch in the hall (EBI 310 TH, Ingolstadt, Germany). These indicators were not statistically significantly different on individual study dates.

All of the players participated in the training throughout its duration. At that time, their heart rate (HR) was monitored using the Polar Team2 PRO Heart Rate Monitoring System (Kempele, Finland) (Table 1). In all competitors, apart from the indicators measured in blood and urine, body mass and the amount of fluids consumed were also monitored before and after training. The complete study scheme is presented in Figure 1.

### 2.6. Statistical Analysis

Data are presented using the mean and standard deviation ($\bar{X}$ ± SD) and the confidence interval for the mean (95% CI). The values of the studied indices were statistically analyzed, and the variables were checked for normal distribution using the Shapiro–Wilk test. In order to compare the pre-training and post-training results obtained between the three study dates, repeated measures analysis of variance (ANOVA) was performed for normally distributed data, and Friedman ANOVA for indices without normal distribution. In order to compare the differences between the examined indices before and after training, the *t*-test for dependent samples was used for data with a normal distribution on individual test dates, and the Wilcoxon pair order test for data without normal distribution. Effect sizes (d) were calculated using means and standard deviations. To determine the effect size, Cohen’s criteria were used [19], which say that values ≥ 0.2 and <0.5 are considered “small”, ≥0.5 and <0.8 “medium”, and ≥0.8 “large”. The level of significance was set at *p* < 0.05. Statistical analysis was performed using a computer statistical package STATISTICA v13.1 (StatSoft, Inc., Tulsa, OK, USA).

## 3. Results

The resting values of the biochemical blood and urine indices of the players on the three study dates did not differ significantly from one another, which proves the homogeneity of the group in terms of the determined indicators. On the other hand, post-exercise differences between individual test terms were found. They concerned plasma osmolality, and the concentration of sodium and potassium ions and aldosterone (Table 4).

The comparison of the values of the examined indicators measured before and after exercise also showed statistically significant differences. They were related to all beverages (low-mineralized water, high-mineralized water, and isotonic drinks) and the indicators determined after their consumption: body mass, hematocrit value, concentration of calcium ions, aldosterone, bicarbonate ions, standard base excess, lactate and urine pH. In the case of consuming water, both low- and high-mineralized, differences in values before and after training were also observed for urine-specific gravity, potassium ion concentration and blood pH. However, the indicators that changed only when consuming isotonic drinks were plasma osmolality, and the concentrations of sodium and chloride ions (Table 4).

## 4. Discussion

This is the first time that such extensive research describing post-training changes in water–electrolyte and acid–base balance in female field hockey players has been presented. Few publications have so far described the effects of fluids with heterogeneous mineral composition and different osmolarity on biochemical blood and urine parameters, adopted by the athletes, especially in women. Therefore, the authors decided to investigate this topic, including in the scope of their research a much wider range of indicators characterizing the water–electrolyte and acid–base balance than in other studies.

The values of the examined indicators, determined before and after the training unit, were in line with the ranges given in previous publications on the impact of exercise on the body. They describe post-exercise reductions in a number of indicators, including body mass [20,21], hematocrit value [22], osmolality [23], the concentration of sodium [21], potassium [24], calcium ions [24,25], magnesium [26], bicarbonate ions [24,27,28], standard base excess [8,28] and blood pH [27,28,29]. An increase in the value after exercise, in our own studies, was observed for urine-specific gravity [30], and the concentrations of aldosterone [31,32] and lactate [24,27,28,29]. However, there are no publications with results that can be related to the data on post-exercise blood chloride concentration and urine pH value in the presented study. It can therefore be assumed that the post-training changes found in the studied female field hockey players do not differ from the data found in other sports disciplines, such as beach volleyball [20], rugby [21,23,27], football [22,24], soccer [29], and basketball [25,30], as well as in swimming exercise [26] and in healthy untrained people after exercise on a treadmill [8] or an ergocyclometer [31,32]. Moreover, changes in the tested biochemical indices in the blood and urine are the result of physical exertion and the accompanying dehydration of the body.

The main question of the research, however, concerned the effect of the osmolarity of the fluids consumed by the athletes during exercise. The proposed arrangement of tests and assays performed for blood and urine biochemical indices made it possible to determine the hydration level of the players before the start of training, which was statistically the same at all times. Additionally, the monitoring of air temperature and humidity ensured that the ambient conditions did not affect the results of the experiment. The average values of air temperature in individual periods were, respectively, 20.9 ± 0.1 vs. 21.0 ± 0.1 vs. 20.8 ± 0.1, and air humidity was 52.5 ± 0.6 vs. 51.0 ± 0.8 vs. 52.0 ± 0.8. Moreover, the players, despite independently making decisions about the amount of fluids consumed during training, adopted a similar volume of experimental drinks on all test dates (Table 1). The above is also visible in the similar mass of urine output after training (Table 1). Such a lack of differences in the amount of urine output, up to 1 h after training, despite the use of fluids with different osmolarity levels, was recently demonstrated by Pence and Bloomer [33]. Their observations also show that drinking water increases urine output 2 to 4 h after drinking it, compared to drinks with a higher content of electrolytes.

The only statistically significant differences between liquids of different osmolarity levels were observed between biochemical markers after drinking water (regardless of the content of minerals in it) and isotonic drinks. These differences are expressed using indicators describing the water–electrolyte balance, such as plasma osmolality, the concentration of sodium and potassium ions, and aldosterone. No statistical differences were observed in the indicators characterizing the acid–base balance. The consumption of an isotonic drink that is rich in sodium caused the smallest increase in the concentration of aldosterone, which is responsible for the reabsorption of this element in the renal tubules, increasing the excretion of potassium ions in the urine [34,35]. As a consequence, the greatest post-exercise reduction in sodium ions and the lowest potassium ions were observed, which also translated into the highest reduction in blood osmolality. Moreover, the concentration of potassium ions measured post-exercise decreased the most in the plasma of players in the case of high-mineralized water, where the highest increase in aldosterone concentration was also noted (Table 4). The small number of publications on this issue and the heterogeneity of the data included therein do not favor a detailed analysis of the issue, especially in the absence of relevant data characterizing players in team games. The ambiguity of the results (Table 5) indicates the need for a more complete study of this issue. All authors analyzing plasma osmolality [36,37,38] and sodium and potassium ions [36,37] blood concentration after consuming fluids with different contents of minerals did not show a statistically significant difference between these fluids. This is in contrast to our research, wherein an isotonic drink was shown to decrease plasma osmolality and blood sodium ions concentration simultaneously, increasing the blood concentration of potassium more effectively than the analyzed waters. Among the publications presented in Table 5, only Powers et al. [36] examined the effect of the used fluids on the blood hydrogen ions’ concentration. Although in our research we did not analyze the concentration of these ions, we did determine the pH values, which are coherent. Powers et al. [36] showed that the consumption of beverages containing electrolytes (EP, GP) more effectively stabilizes the concentration of hydrogen ions in the blood than liquids without electrolytes (NEP), especially during exercise. In our study, we did not observe any differences in the acid–base balance indicators determined in blood and urine, regardless of the used fluids. Due to the fact that we have the opportunity to compare our results only with one study [36], we are not able to clearly explain the reasons for the differences. This requires research involving a larger number of participants, as well as efforts (training loads) of varying intensity.

However, our own research shows that the amount of fluids, but not the quality, is of greater importance for maintaining the correct water–electrolyte and acid–base balance.

Our study has some limitations, which include the lack of the consideration of ions in the urine and sweat. Analyses of the 24 h diet diaries before the performed tests were also not undertaken.

The essential point of this manuscript is that the research topic is related to the determination of the effect of fluids with different minerals contents on the water–electrolyte and acid–base balances. The available literature on this topic does not have homogeneous results and specific recommendations for hydration strategies in various sports disciplines, including field hockey. It was also the first time that such a wide range of blood and urine biochemical parameters was used. The benefit of this study is that the measurements were carried out in real training conditions, and not directly in isolated laboratory tests.

In the future, in order to more accurately assess the aim set in the study, we plan to repeat the research, increasing the number of female players, including testing players from other team games.

## 5. Conclusions

Based on a review of the available literature, we found that field hockey does not differ from other sports in terms of the biochemical blood and urine indicators characterizing the post-training changes of players.

The osmolarity of consumed fluids does not significantly affect the indicators of the water–electrolyte balance and acid–base balance during exercise. Such an effect is only noticeable after consuming an isotonic drink, manifesting itself in greater changes in the concentration of aldosterone, sodium and potassium ions and plasma osmolality than in the case of hypotonic drinks. Furthermore, the degree of mineralization of the water consumed by female field hockey players did not affect the indicators of water–electrolyte and acid–base balance in the blood and urine.

Isotonic drinks, unlike hypotonic drinks, most likely stabilize the RAA system during training, which ensures the best hydration as defined by plasma osmolality.

The wide spectrum of commercially available sports drinks and waters used by athletes raises the question of selecting those liquids that stabilize the water–electrolyte and acid–base balances. Moreover, they should positively affect the exercise capacity of athletes. The information contained in this publication discusses this issue in terms of the different osmolarity levels of beverages, making the applied knowledge useful for both players and coaches.

## Figures and Tables

**Table 1:** Somatic and physiological characteristics of female field hockey players (*n* = 14).

Characteristics	Low-Mineralized Water		High-Mineralized Water		Isotonic Drink		*p* Value
	$\bar{X}$ ± SD	95% CI	$\bar{X}$ ± SD	95% CI	$\bar{X}$ ± SD	95% CI	
Age (years)	21.9 ± 2.3	(20.6–23.2)	21.9 ± 2.3	(20.6–23.2)	21.9 ± 2.3	(20.6–23.2)	1.000
Body height (m)	1.70 ± 0.06	(1.67–1.74)	1.70 ± 0.06	(1.67–1.74)	1.70 ± 0.06	(1.67–1.74)	1.000
Body mass (kg)	65.3 ± 5.4	(62.2–68.4)	65.4 ± 5.4	(62.2–68.5)	65.2 ± 5.0	(62.3–68.1)	0.712
WHtR	42.9 ± 1.4	(42.1–43.7)	43.0 ± 1.4	(42.1–43.8)	42.9 ± 1.4	(42.1–43.7)	0.906
HR mean (bpm)	151.7 ± 3.0	(149.9–153.4)	152.0 ± 4.3	(149.5–154.5)	151.6 ± 2.1	(150.4–152.9)	0.962
Fluid intake (ml)	543.9 ± 270.0	(388.0–699.8)	535.7 ± 180.2	(431.7–639.8)	503.6 ± 224.7	(373.9–633.3)	0.800
Urine mass excreted after training (g)	157.1 ± 70.4	(116.5–197.8)	135.0 ± 104.6	(74.6–195.4)	115.7 ± 72.8	(73.7–157.8)	0.404

WHtR: waist-to-height ratio; HR: heart rate; $\bar{X}$: average; SD: standard deviation; CI: confidence interval.

**Table 2:** Framework training unit plan.

Training Group	Women’s National Team and Women’s Junior Team
**Training duration**	90 min
**Venue**	Indoor Hall 40 m × 20 m
**Training objective**	Preparation for indoor championship events according to the calendar of the European Field Hockey Federation
**Warm-up**	Warm-up incl. dynamic stretching + acceleration and speed drills—15 min Hockey-specific warm-up: various forms of passing and receiving the ball in motion (without the participation of a defender); shorter and longer passes, also with the use of a boards—5 min Scoring exercises (different zones of the shooting circle)—5 min
**Training**	Numerical advantage training—2 vs. 1 and 3 vs. 2/defensive organization in the numerical superiority of the opponent; cooperation with the goalkeeper—20 min Tactical cooperation in even numbers situation—3 vs. 3 on the side sector of the pitch (left and right board) with an emphasis on the transition phase (transition from defending to attacking)—15 min Build-up in 5 vs 4 superiority—4 × 3 min + 1 min break after every 3 min 5 vs. 4 game—2 × 5 min (change of teams after 5 min)

**Table 3:** The mineral composition of the fluids, specified by the manufacturer, consumed by female field hockey players during training.

Mineral	Low-Mineralized Water	High-Mineralized Water	Isotonic Drink + Low-Mineralized Water
	(mg/L)		
Ca^2+^	48.10	319.00	288.10
Na^+^	2.10	111.00	702.10
Mg^2+^	6.68	47.90	126.68
K^+^	1.20	49.50	261.20
HCO_3_^−^	166.30	1639.00	166.30
SO_4_^2−^	10.29	30.00	10.29
Cl^−^	5.60	2.70	245.60
F^−^	0.06	0.30	0.06
Total minerals	240.33	2199.40	1800.33
Glucose	0.00	0.00	52,600.00

**Table 4:** Average values of the tested biochemical indicators of blood and urine in the examined persons after consuming beverages with different osmolarity levels (*n* = 14).

Indicator	Beverages	Pre-Exercise	Post-Exercise	*p* Value (Pre vs. Post)	Effect Size	*p* Value for ANOVA (Post-Exercise Differences between Beverages)
Body mass (kg)	Low	65.3 ± 5.4	65.1 ± 5.4	0.002	0.04	0.706
	High	65.4 ± 5.4	65.2 ± 5.5	0.001	0.03	
	Isotonic	65.2 ± 5.0	65.0 ± 5.0	<0.001	0.04	
**Water Balance**						
Hematocrit (l/L)	Low	0.377 ± 0.017	0.366 ± 0.021	0.048	0.56	0.212
	High	0.372 ± 0.020	0.360 ± 0.023	<0.001	0.59	
	Isotonic	0.367 ± 0.020	0.353 ± 0.021	<0.001	0.71	
Urine specific gravity (g/L)	Low	1.013 ± 0.006	1.019 ± 0.008	0.001	0.82	0.108
	High	1.014 ± 0.006	1.023 ± 0.009	0.006	1.15	
	Isotonic	1.016 ± 0.009	1.019 ± 0.007	0.068		
Plasma osmolality (mOsm/kg)	Low	291.5 ± 2.1	290.1 ± 3.6	0.155		<0.001 ^a^
	High	291.6 ± 2.5	290.1 ± 3.6	0.077		
	Isotonic	290.1 ± 2.9	285.3 ± 2.6	0.001	1.73	
**Electrolyte Balance**						
Sodium ions (mmol/L)	Low	143 ± 1	142 ± 2	0.111		0.005 ^a^
	High	143 ± 1	142 ± 2	0.179		
	Isotonic	142 ± 2	140 ± 1	<0.001	1.51	
Potassium ions (mmol/L)	Low	4.4 ± 0.4	4.1 ± 0.4	0.024	0.66	0.022 ^b^
	High	4.3 ± 0.3	3.9 ± 0.3	<0.001	1.39	
	Isotonic	4.5 ± 0.4	4.3 ± 0.3	0.075		
Calcium ions (mmol/L)	Low	1.21 ± 0.03	1.19 ± 0.03	0.031	0.76	0.624
	High	1.20 ± 0.02	1.18 ± 0.04	0.030	0.60	
	Isotonic	1.22 ± 0.03	1.19 ± 0.03	<0.001	1.06	
Chloride ions (mmol/L)	Low	109 ± 1	108 ± 2	0.418		0.357
	High	108 ± 2	107 ± 2	0.292		
	Isotonic	109 ± 2	107 ± 2	<0.001	0.80	
Magnesium (mmol/L)	Low	0.89 ± 0.01	0.89 ± 0.03	0.730		0.789
	High	0.89 ± 0.01	0.89 ± 0.02	0.431		
	Isotonic	0.89 ± 0.02	0.90 ± 0.02	0.272		
Aldosterone (pmol/L)	Low	125.8 ± 45.4	411.8 ± 184.0	0.001	2.13	0.005 ^a^
	High	117.0 ± 53.9	424.2 ± 107.5	<0.001	3.61	
	Isotonic	112.2 ± 28.4	270.2 ± 104.6	<0.001	2.06	
**Acid–Base Balance**						
Bicarbonate ions (mmol/L)	Low	24.1 ± 1.6	22.4 ± 1.3	<0.001	1.13	0.683
	High	24.7 ± 2.1	22.8 ± 1.5	0.005	1.05	
	Isotonic	24.3 ± 1.4	22.3 ± 2.0	<0.001	1.16	
Standard base excess (mmol/L)	Low	−0.1 ± 1.3	−2.6 ± 1.8	<0.001	1.63	0.645
	High	0.9 ± 1.7	−2.1 ± 2.0	<0.001	1.61	
	Isotonic	0.1 ± 1.7	−2.8 ± 2.8	<0.001	1.26	
Blood pH	Low	7.40 ± 0.03	7.39 ± 0.03	0.024	0.45	0.926
	High	7.41 ± 0.02	7.39 ± 0.03	0.012	0.82	
	Isotonic	7.40 ± 0.03	7.39 ± 0.03	0.102		
Urine pH	Low	6.2 ± 0.7	5.4 ± 0.6	0.002	1.31	0.313
	High	6.2 ± 0.7	5.6 ± 0.6	0.005	0.89	
	Isotonic	6.0 ± 0.7	5.5 ± 0.6	0.018	0.81	
Lactate (mmol/L)	Low	1.3 ± 0.4	5.8 ± 1.7	0.001	3.64	0.807
	High	1.3 ± 0.3	5.9 ± 2.6	0.001	2.54	
	Isotonic	1.4 ± 0.4	5.9 ± 2.5	0.001	2.55	

Low—low-mineralized water, High—high-mineralized water, Isotonic—sport drink; ^a^—the average values for isotonic drinks are lower than for both waters; ^b^—the average value for isotonic water is higher than for high-mineralized water.

**Table 5:** Summary of publications on the impacts of drinks with different (indicated by the authors of the studies) osmolarity levels on the biochemical and hematological indicators of people subjected to exercise tests.

Authors (Sport Discipline) (Kind of Effort) Sex	The Types of Beverages	Tested Biochemical Indicators	
		No Significant Differences	Significant Differences
Powers et al. [36] (cyclists; *n* = 9) (exercises with a constant load on a bicycle ergometer until fatigue) Men	Non-electrolyte placebo (NEP) (31 mOsm/kg)	Heart rate, plasma osmolality, concentration of lactate, potassium, calcium, sodium, and chloride in blood	The concentration of hydrogen ions in the blood was significantly lower after 30 min of exercise while using GP and EP compared to NEP
	Electrolyte placebo drink without carbohydrate (EP) (48 mOsm/kg)		
	Glucose polymer drink containing electrolytes (GP) (231 mOsm/kg)		
Gisolfi et al. [37] (wytrenowani; *n* = 7) (85 min 60%–65% VO_2_ma xcycle ergometer) 5 Men, 2 Women	Water (1 ± 0.3 mOsm/kg)	Osmolarity, sodium and potassium ions in plasma	There are no statistically significant differences
	Hypertonic (197 ± 2 mOsm/kg)		
	Isotonic (295 ± 6 mOsm/kg)		
	Hypotonic (414 ± 2 mOsm/kg)		
Suzuki et al. [38] (cyclists; *n* = 6) (cycling at 60% VO_2_peak for 90 min in the hot conditions) Men	Plain water (no data)	Plasma osmolality, lactate concentration	There are no statistically significant differences
	Hypotonic sports drink (193 mOsm/kg)		
	Isotonic sports drink (317 mOsm/kg)		
Łagowska et al. [39] (rowers; *n* = 11) (80 min of exercises on a rowing ergometer) Men	Commercially available sports drink (258 mOsm/kg)	Lactate concentration, hematocrit	There are no statistically significant differences
	Natural carbohydrate electrolyte drink (402 mOsm/kg)		
Our work (field hockey; *n* = 14) (90-min training unit) Women	Low-mineralized water (~20 mOsm/kg)	HR, hematocrit, concentration of lactate, calcium, chloride and bicarbonate ions, magnesium, standard base excess, blood and urine pH, and urine-specific gravity	Consumption of an isotonic drink caused the smallest increase in the concentration of aldosterone and potassium ions, and the greatest post-exercise reduction in sodium ions and blood osmolality
	High-mineralized water (~88 mOsm/kg)		
	Isotonic drink (~279 mOsm/kg)		

VO_2_max: maximal oxygen uptake; VO_2_peak: peak oxygen uptake.

## Data Availability

The data presented in this study are available on request from the corresponding author. The data are not publicly available due to ethical restrictions.
